# Performance Analysis of a Fiber Reinforced Plastic Oil Cooler Cover Considering the Anisotropic Behavior of the Fiber Reinforced PA66

**DOI:** 10.3390/polym8090312

**Published:** 2016-09-01

**Authors:** Jian Wang, Junhong Zhang, Jiewei Lin, Weidong Li, Huwei Dai, Huan Hu

**Affiliations:** 1 State Key Laboratory of Engines, Tianjin University, Tianjin 300072, China; wangjian11@tju.edu.cn (J.W.); liwd@tju.edu.cn (W.L.); dhwmail@tju.edu.cn (H.D.); huhuan0804@tju.edu.cn (H.H.); 2 Department of Mechanical Engineering, Tianjin University Renai College, Tianjin 301636, China

**Keywords:** orthogonal anisotropic, glass fiber reinforced PA66, diesel engine oil cooler cover, lightweight, finite element method

## Abstract

In this paper, a simulation method based on an orthogonal anisotropic material is proposed. A numerical example using a simple plate is presented to show the difference in the static performance between the orthogonal anisotropic and the isotropic models. Comparing with the tested modal data of a diesel engine oil cooler cover made by glass fiber reinforced polyamide 66 (PA66), the proposed simulation method was confirmed to be much closer to reality than the general isotropic model. After that, a comprehensive performance comparison between the plastic oil cooler covers with the orthogonal anisotropic and the isotropic fiber orientations was carried out including a static deformation and stress analysis under a pressure-temperature coupled load, a forced response analysis, and an acoustic analysis under real operating conditions. The results show that the stress, the deformation, the peak vibration velocity, and the overall sound power level of the orthogonal anisotropic model are different from that obtained with the isotropic model. More importantly, the proposed method can provide a much more detailed frequency content compared to the isotropic model.

## 1. Introduction

Glass fiber reinforced thermoplastics are increasingly used in manufacturing [1,2,3]. Compared with metal materials, glass fiber reinforced thermoplastics offer considerable advantages including lower density, higher damping, stronger corrosion resistance, and better manufacturing capability [4,5,6]. In addition, glass fiber reinforced thermoplastics provide greater mechanical performance and temperature resistance than common plastics. Therefore more and more glass fiber reinforced plastic components are being used to replace those made of traditional materials in the contemporary automobile industry in order to pursue lower manufacture cost, lighter weight, and higher fuel efficiency. Furthermore, the recyclability, which is rapidly being recognized as important, is another strong driving force for the further application of fiber reinforced thermoplastics.

The mechanical performance of the glass fiber reinforced plastic component strongly depends on the proportion of the glass fiber. Additionally, fiber orientation is another important factor affecting mechanical performance. Fiber orientation can be influenced by component geometry and the injection process, such as the injection position, the number and timing of the injection gates, the injection pressure and time, the mold temperature, and the melt temperature [7,8,9]. The mechanical properties along the fiber are very different from those in the normal direction, which means the glass fiber reinforced plastic is a typical anisotropic material.

Experimental studies are the first choice to investigate the characteristics of anisotropic plastics. Thomason [10,11,12] studied the effects of the fiber length, the content, and the fiber diameter on glass fiber reinforced polyamide 66. The mechanical properties were found to improve with increasing glass content, and the impact showed an initial decrease from the resin value with a minimum at 4% glass content before increasing to higher glass contents. The composite tensile strength was reduced by broadening the fiber diameter distribution compared to an average diameter. Bernasconi et al. [13] investigated the effect of mechanical recycling upon tensile strength of an injection molded polyamide 66 reinforced with 35% by weight of glass fibers. The test results showed a decrease of both elastic modulus and tensile strength with increasing content of reprocessed material. The effects of the fiber volume fraction, length, and orientation on the tensile behavior of short fiber reinforced polyamide were also analyzed [14]. In addition, an experimental study of the fatigue strength was conducted to study the combined effect of the notch tip radius and the injection gate position on the injection molded specimens [15]. Wang et al. [16] tested tensile properties of short glass fiber reinforced polyamide-6 in two different directions at different strain rates and temperatures. The tensile strength and the elastic modulus in the extrusion direction are about twice as large as those in the direction normal to the extrusion. Zhou and Mallick [17] carried out an experimental study on the mechanical properties of short glass fiber reinforced PA66 at different strain rates. The tensile strength and the elastic modulus normal to the flow direction decreased by 35% and 43% respectively compared with those in the flow direction. De Monte et al. [18] studied the anisotropic behavior of the mechanical properties of short glass fiber reinforced polyamide 66 (PA66-GF35) under quasi-static loading. Tensile tests were carried out with dog-bone specimens, which were machined from injection molded plates of three different thicknesses (1–3 mm) at eight different orientation angles. The tensile strength and the elastic modulus decreased as the orientation angle increases. A marked anisotropy could be noticed at a thickness of 1 mm, which gradually reduced with increasing thickness. Mortazavian and Fatemi [19] conducted an experiment to investigate anisotropy effects on the tensile properties of two short glass fiber reinforced thermoplastics (PA6 and PBT). The 45° direction samples exhibited nearly the same tensile strength and elastic modulus as the transverse direction ones, which were significantly lower than that in the longitudinal direction. In addition, the 45° direction samples indicated a higher ductility compared with the longitudinal and transverse directions. Apart from the mechanical properties of the glass fiber reinforced thermoplastics, fiber orientation also influences the thermal behavior of the material. Tezvergil et al. [20] analyzed the effect of fiber orientation on the thermal expansion coefficients of fiber-reinforced composites. The results showed that the thermal expansion in the longitudinal fiber direction is smaller than that in the transversal direction. Heinle and Drummer [21] proposed a method to calculate the temperature-dependent anisotropic coefficient of the thermal expansion of short fiber reinforced polymers. The computed results agreed well with the experiment which means the modelling method is able to predict the anisotropy of the thermal expansion coefficient correctly.

In terms of manufacturing, the simulation approach plays an important role particularly in the prototype design phase. At the moment, modeling studies on anisotropic materials range from short fiber composite materials researches and bone researches to the building structure field. Ding et al. [22,23,24] constructed a mandible model and assigned mandibular bone to the orthotropic material characteristics. The influence of orthotropic material on the biomechanical behavior of a complete dentate mandible was analyzed and compared with a commonly used isotropic model. The result revealed that the orthotropic model would induce higher stress and a more well-distributed stress pattern than the isotropic model. Taylor et al. [25] used computed tomography (CT) to scan a cadaveric bone and constructed the geometry. The values of the orthotropic elastic constants were then established by matching the predictions from Finite element (FE) modal analyses to the experimental natural frequencies. Finally, the elastic constants of the bone derived from the analyses were compared with those measured using ultrasound techniques, and the differences were lower than 1% for both the maximum density and the axial Young’s Modulus. Researchers have also been using a combination of the manufacturing simulation output [26] (2nd order orientation tensor) and the inclusion based models [27] (Mori-Tanaka model) for simulating the behavior of short fiber composite materials. Jain et al. [28] opposed the use of pseudo-grain discretization and showed the advantages of Mori-Tanaka formulation [27]. Also, a method of treatment of inclusions with deboned interface by replacing them with a fictitious “equivalent bonded inclusion” was proposed [29]. Additionally, a hybrid (combination of micromechanics and tests) and a multi-scale (damage in micro-scale linked to macroscale fatigue properties) method of predicting the SN curve for every point in a short fiber composite have been developed [30]. Meraghni et al. [31] modelled the effect of matrix degradation on the overall behavior of randomly oriented discontinuous-fiber composites and a micromechanical analysis based on the modified Mori-Tanaka model was performed. Brighenti et al. [32] adopted an energy-based homogenization approach to model the mechanical behavior of fiber-reinforced materials for obtaining the macro constitutive equations used in the numerical modeling of the fracture behavior of brittle materials reinforced with unidirectional or randomly distributed fibers [33]. Zhang et al. [34] proposed a multiple scale modeling and simulation scheme based on an equivalent orthotropic material modeling method. The static and dynamic responses and the dynamic properties of a simplified short span bridge from the model were obtained. Based on the modeling scheme, it is possible to predict reasonable static or dynamic responses of the bridge details. Furthermore, some studies on simulation analysis of fiber reinforced PA66 component have also been carried out. Bikard et al. [35] predicted the deformation of a hybrid beam with short fiber reinforced PA66 material. Injection simulation was performed using Moldflow software to obtain the orientation tensor field, and the structural simulation was conducted using Ansys and Digimat software. Mouti et al. [36,37] investigated the localized low velocity impact on glass fiber reinforced polyamide oil pan in typical flying stones impact scenarios. An FE analysis using LS DYNA (Livermore Software Technology Corporation (LSTC), Las Vegas, NV, USA) was carried out to predict the strength and the fracture behavior of the pressure-containing plastic parts. The reliability of the simulation was validated by comparing the simulation results to the experiment data.

Since the mechanical performance of fiber reinforced plastics varies with fiber orientation and structure geometry, simulations using isotropic and anisotropic materials would give very different results both in static analysis and in dynamic analysis. In practice, the material used to produce the fiber reinforce plastic components is anisotropic, which means the simulation based on the isotropic material definition might introduce significant errors, especially for a plate shaped component with long fiber. Moreover, a misleading direction in structural optimization would be obtained on the basis of an inaccurate analysis of results. For instance, a deviated modal frequency may result in a structural resonance if the component is integrated into a larger system, which would lead to an adverse effect on system performance.

As a part of a diesel engine, the oil cooler cover operates under alternating pressure, temperature, and vibration loads. So the dynamic responses of the oil cooler cover under the complex loading condition needs to be carefully considered in addition to its mechanical properties. In this study, an orthogonal anisotropic material simulation method was proposed and applied to a plastic oil cooler cover made of glass fiber reinforced PA66 in order to find out the effect of the orthotropic characteristics on the mechanical, thermal, vibration, and acoustic behaviors of the components. By calculating the material properties from the elastic constitutive model, the performance of a rectangular plate made of anisotropic material was compared with that made of isotropic material. Then the FE model of the oil cooler cover and its mold injection model while considering orthogonal anisotropic behavior were developed and validated by the experimental modal data. According to the loading conditions obtained from the computational fluid dynamics (CFD) calculation, the static and dynamic performances of the oil cooler cover were compared with that of the isotropic material and discussed in detail.

## 2. Elastic Constitutive Model of the Orthogonal Anisotropic Material

An orthotropic material has three mutually perpendicular axes of symmetry at every point, and the material properties differ along these orthotropic directions [38]. The uncoupled stress–strain relationship can be defined based on the generalized Hooke’s law [39]. A transversely isotropic material has symmetric properties about the axis normal to a plane of isotropy [40]. The material properties are the same in all directions within the transverse plane. The unidirectional composite material is transversely isotropic and the plane normal to the fiber orientation can be considered as the transverse plane. Let the axis along the fiber orientation be the *x*_1_ direction, and the other two axes on the transvers plane be the *x*_2_ and *x*_3_ directions. The flexibility matrix can be given as:

(1) $${\left[C\right]}_{m}=\left[\begin{array}{cccccc} 1/E_{1} & -\mu_{12}/E_{1} & -\mu_{12}/E_{1} & 0 & 0 & 0\\ -\mu_{12}/E_{1} & 1/E_{2} & -\mu_{23}/E_{2} & 0 & 0 & 0\\ -\mu_{12}/E_{1} & -\mu_{23}/E_{2} & 1/E_{2} & 0 & 0 & 0\\ 0 & 0 & 0 & 1/G_{23} & 0 & 0\\ 0 & 0 & 0 & 0 & 1/G_{12} & 0\\ 0 & 0 & 0 & 0 & 0 & 1/G_{12} \end{array}\right]$$

A more detailed derivation process can be found in the Appendix.

## 3. Comparison between Isotropic and Orthogonal Anisotropic Materials

### 3.1. Model and Material Properties

**Figure 1:**
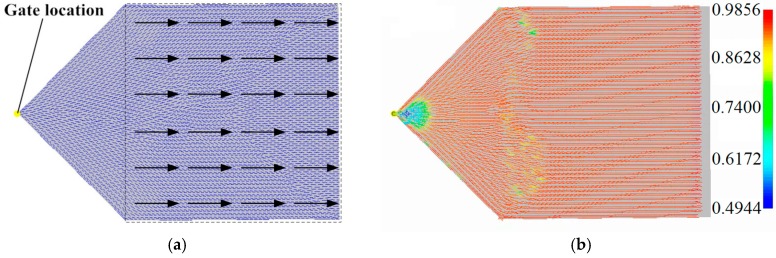
Fiber orientations of the transversely isotropic material plate (dashed region), (**a**) the fiber orientation and (**b**) the corresponding fiber orientation tensor (a11).

Due to the material properties of the orthogonal anisotropic component varying according to the fiber orientation, a Moldflow analysis (Autodesk, San Rafael, CA, USA)) was conducted to obtain the fiber orientation of the plate sample so that the material properties of the FE model of the plate could be allocated accordingly. To compare the orthogonal anisotropic material to the isotropic one, a square plate (500 mm × 500 mm × 5 mm) model of the unidirectional composite material was constructed as shown in Figure 1. The arrows show the directions of the injection flow, and the dashed region shows the fiber orientation distribution of the plate studied in this section. The material used is a type of PA66 with 30% fiber glass (GF) supplied by Lanxess Chemical Co. Ltd. (Shanghai, China). The properties of the PA66 (GF 30%) are listed in Table 1. The location of the injection mold gate was in the middle, which makes the fiber orientation uniform.

Then the flexibility matrix [*C*]*_m_* and the stiffness matrix [*D*]*_m_* of the transversely isotropic material can be obtained as Equations (2) and (3).

The isotropic material properties are given as *E* = 8,500 MPa, µ = 0.4, *G* = 3,036 MPa, and *α* = 6 × 10^−5^ 1/K, which are supplied by the Lanxess Chemical Co. Ltd.

(2) $${\left[C\right]}_{m}=0.001*\left[\begin{array}{cccccc} 0.0971 & -0.0388 & -0.0388 & 0 & 0 & 0\\ -0.0388 & 0.1471 & -0.0588 & 0 & 0 & 0\\ -0.0388 & -0.0588 & 0.1471 & 0 & 0 & 0\\ 0 & 0 & 0 & 0.4118 & 0 & 0\\ 0 & 0 & 0 & 0 & 0.5236 & 0\\ 0 & 0 & 0 & 0 & 0 & 0.5236 \end{array}\right]\;(1/\mathrm{MPa})$$

(3) $${\left[D\right]}_{m}=10000*\left[\begin{array}{cccccc} 1.5898 & 0.6997 & 0.6997 & 0 & 0 & 0\\ 0.6997 & 1.1175 & 0.6318 & 0 & 0 & 0\\ 0.6997 & 0.6318 & 1.1175 & 0 & 0 & 0\\ 0 & 0 & 0 & 0.2429 & 0 & 0\\ 0 & 0 & 0 & 0 & 0.1910 & 0\\ 0 & 0 & 0 & 0 & 0 & 0.1910 \end{array}\right]\;(\mathrm{MPa})$$

### 3.2. Comparison between Different Materials

**Figure 2:**
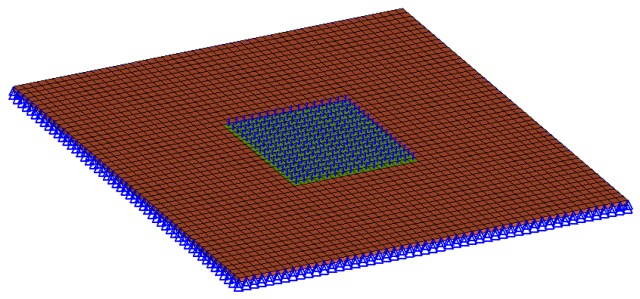
Boundary and loading conditions of the square plate model.

The FE model of the plate consists of 2,500 hexahedron elements. In the simulation, all six degrees of freedom (DOFs) of the nodes on the four edges were constrained and an evenly distributed pressure of 0.1 MPa was applied on the center square area of the plate as marked in blue (shown in Figure 2). The linear static analysis of the plate was carried out using MSC Nastran (MSC Software, Los Angeles, CA, USA).

**Figure 3:**
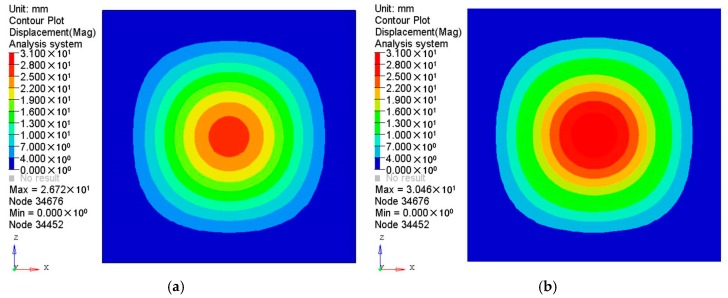
Displacements of (**a**) isotropic and (**b**) orthotropic plates under pressure of 0.1 MPa.

**Figure 4:**
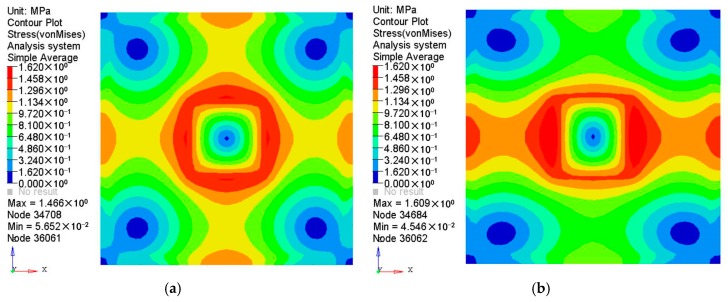
Stress distributions of (**a**) isotropic and (**b**) orthotropic plates under pressure of 0.1 MPa.

The results of the displacement and the stress distributions are shown in Figure 3 and Figure 4 respectively. Seen from Figure 3, the deformation distributions of the two materials are similar since all the edges are fixed. However, the peak deformation of the orthogonal anisotropic plate is larger than that of the isotropic one due to the normal elastic modulus of the former being smaller than that of the latter. Comparing the stress between the two plates (shown in Figure 4), it can be seen that the stress distribution of the isotropic plate is centrally symmetrical and that of the orthogonal anisotropic plate is not. As the principal elastic modulus is larger than the normal modulus, the stress along the principal direction (*x* axis) is larger than that along the vertical direction (*z* axis). The maximum stress of the orthogonal anisotropic plate is about 10% larger than that of the isotropic one.

**Figure 5:**
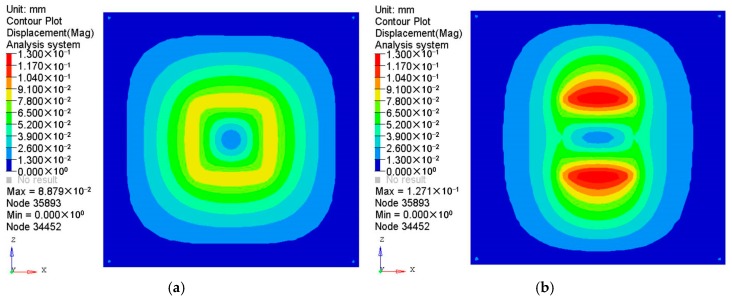
Displacements of (**a**) isotropic and (**b**) orthotropic plates under the temperature difference of 30 K.

**Figure 6:**
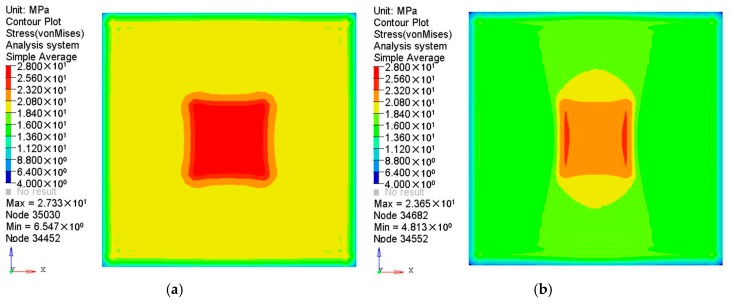
Thermal stress distributions of (**a**) isotropic and (**b**) orthotropic plates under the temperature difference of 30 K.

To analyze the temperature effect on the two materials, the load was changed from pressure to temperature and the constraints remained the same. The temperature of the center square area was set as *T*_1_ = 323 K and that of the rest was set as *T*_2_ = 293 K (room temperature) according to the expected temperature difference of the oil cooler. The thermal deformation and stress results are shown in Figure 5 and Figure 6 respectively. With an isotropic material, the thermal deformation of the square plate is almost centrally symmetrical, but that of the orthogonal anisotropic material shows an axisymmetric distribution. It may be because the thermal expansion coefficient in the *z* direction is higher than that in the *x* direction which could make a higher deformation in the *z* direction than that in the *x* direction with the same distance from the center point. The peak deformation appears at the boundaries between the two temperatures and the deformation attenuates along the radial direction for the isotropic case. In the orthogonal anisotropic case, the maximum deformation appears at the boundaries between the loaded and unloaded areas only in the *z* direction. Besides, the maximum thermal deformation of the isotropic plate is smaller than that of the orthogonal anisotropic plate. In terms of the thermal stress distribution, the difference between the two materials is similar to that of the deformation, but the maximum stress of the isotropic plate is about 16% larger than that of the orthogonal anisotropic plate.

**Figure 7:**
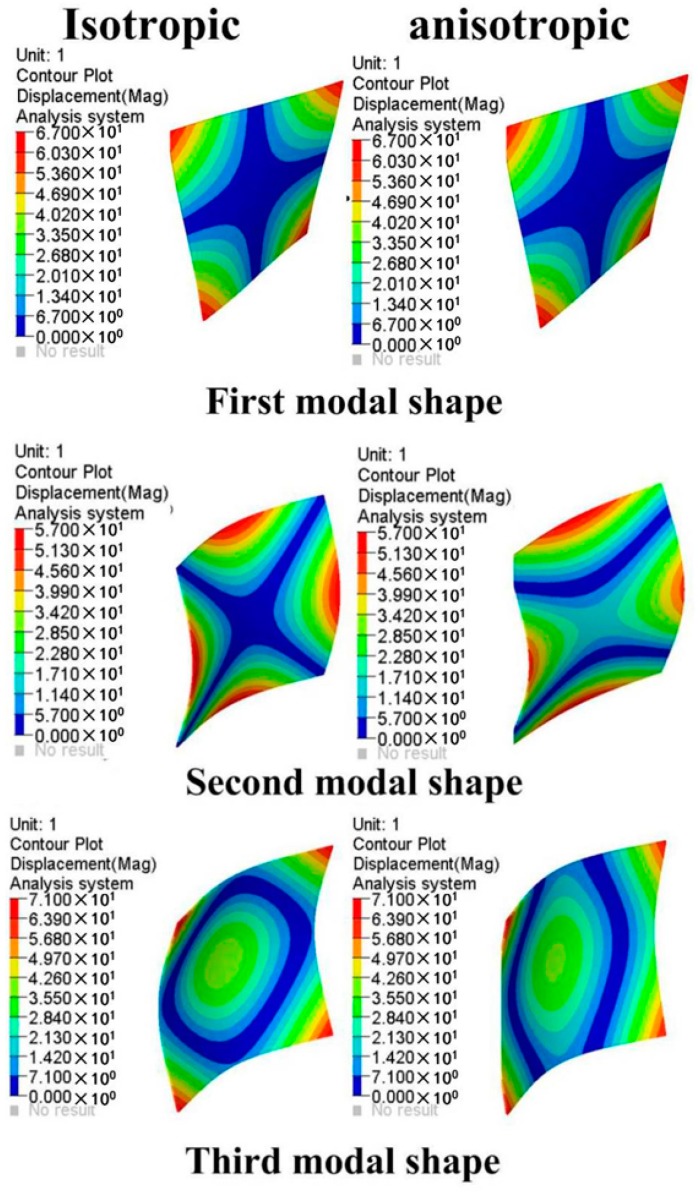
Comparisons of free mode shapes between the isotropic and the anisotropic plates.

To compare vibration characteristics between the two plates, the first five order modal frequencies and the first three order mode shapes were analyzed and are given in Table 2 and Figure 7. It can be seen that every modal frequency of the orthogonal anisotropic plate is correspondingly lower than that of the isotropic plate. As the densities of the two plates are the same, it means the stiffness of the orthogonal anisotropic plate is lower than that of the isotropic plate. In Figure 7, it can be seen that the first three mode shapes of the isotropic plate are all centrally symmetrical which are very different from those of the anisotropic plate especially in the second and the third modes. All the modes of the isotropic plate can be considered as diagonal-symmetrical, but those of the orthogonal anisotropic plate have to be defined as axisymmetric along the *x* or *y* direction apart from the first mode. The first modes of the two plates are almost the same which may be because the difference in the elastic modulus in the principal and the vertical directions does not affect the vibration significantly at low frequencies.

## 4. Performance Analysis of the Plastic Oil Cooler Cover

### 4.1. Model Information

#### 4.1.1. Injection Model

**Figure 8:**
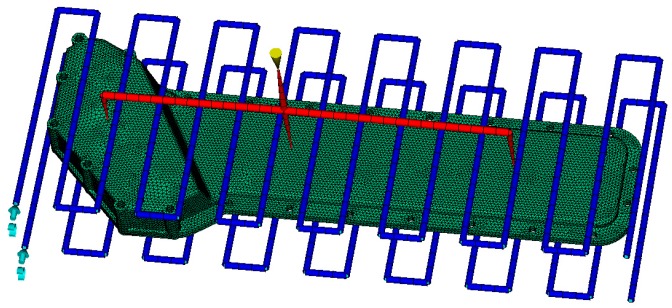
The FE model of the injection molding system of the oil cooler cover.

**Figure 9:**
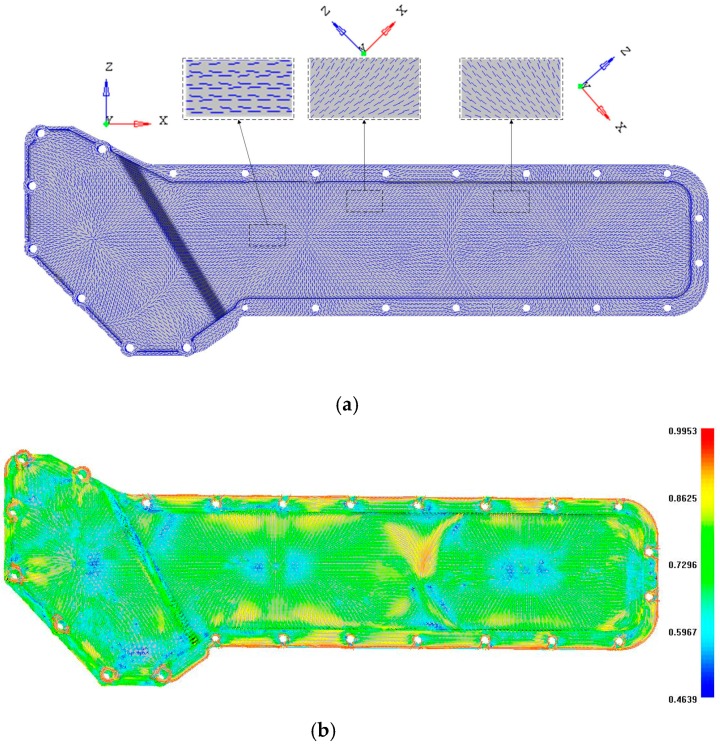
The fiber orientation of the plastic oil cooler cover, (**a**) the fiber orientation and (**b**) the corresponding 2nd order fiber orientation tensor (a11).

The oil cooler cover studied in this paper is a component of a diesel engine. It works under a combined load consisting of pressure and temperature caused by the cooling water and the vibration excitation from the engine body. The size of the oil cooler cover is 873 mm × 304 mm × 64 mm and the thickness is 4 mm. The material used is a type of PA66 with 30% fiber glass (GF), which is the same as the material used in Section 3.1. To obtain a realistic fiber orientation of the plastic oil cooler cover, a FE model of the injection molding system was developed consisting of an oil cooler cover, two cooling channels, and a runner system as shown in Figure 8. The cooling channel diameter is 10 mm. A valve gated hot runner with three gates was employed to control the valve gate timing so as to carry out a sequential injection to reduce the warpage in the injection molding process [41]. The FE model contains 48,725 tetrahedron elements and the injection molding process was simulated using Moldflow (version 2012). The nodes around the screw holes were constrained in all six DOFs and the loads were mapped from the CFD results directly. As shown in Figure 9, the fiber orientation of the plastic oil cooler cover varies in different areas. The local fiber orientation is enlarged as shown as the picture in the wireframe. The corresponding local coordinate systems, namely the orthotropic axes, are also indicated in the figure. The fiber orientation is in the *x* direction.

#### 4.1.2. Fluid Structure Coupled Model

**Figure 10:**
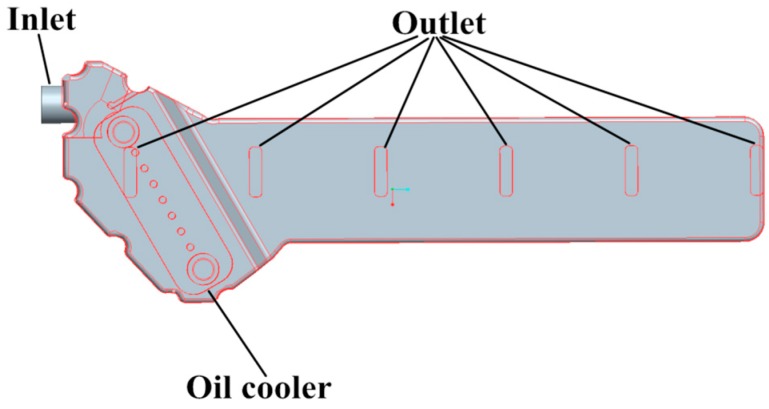
The computational fluid dynamics (CFD) model of the oil cooling system.

As shown in Figure 10, the plastic oil cooler cover is a part of the oil cooling system which contains another two parts: the oil cooler and the water cavity. To obtain the loading conditions of the plastic oil cooler cover, a CFD simulation of the oil cooling system was carried out.

The FE model of the oil cooling system consists of an inlet, six outlets, the walls, and the water cavity. A mass-flow inlet (9 kg/s, and *T*_in_ = 358 K), pressure outlets (*P*_out_ = 0.158 MPa, and *T*_out_ = 363 K), and a stationary wall without slip were used in the analysis. The temperatures of the oil cooler and the cover were set as 373 and 313 K respectively. The water cavity consisted of 1,013,594 fluid elements. The CFD simulation was carried out using the RNG k−ε model and the main parameters used in the calculation are:

Cooling liquid:
  $\rho_{L}=981.43\;\mathrm{kg}/m^{3},\;Cp_{L}=3580\;J/(\mathrm{kg}\cdot K),\;\lambda_{L}=0.3419\;W/(m\cdot K),\;\eta_{L}=0.00051\;\mathrm{kg}/(m\cdot s)$
Gray cast iron:
  $\rho_{L}=7030\;\mathrm{kg}/m^{3},\;Cp_{L}=480\;J/(\mathrm{kg}\cdot K),\;\lambda_{L}=54\;W/(m\cdot K)$
PA66 (GF 30%):
  $\rho_{L}=1380\;\mathrm{kg}/m^{3},\;Cp_{L}=1260\;J/(\mathrm{kg}\cdot K),\;\lambda_{L}=0.52\;W/(m\cdot K)$

**Figure 11:**
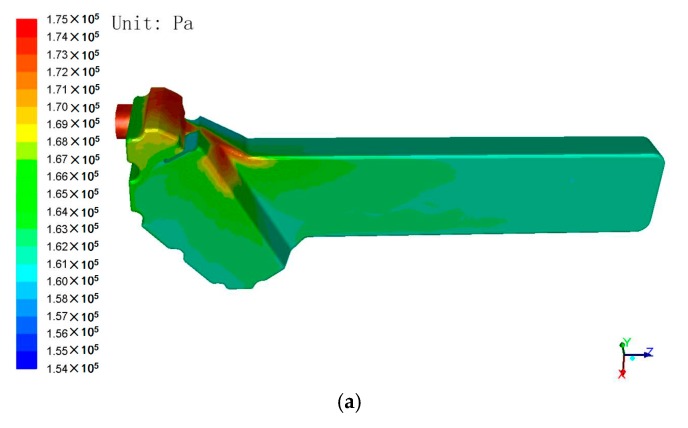

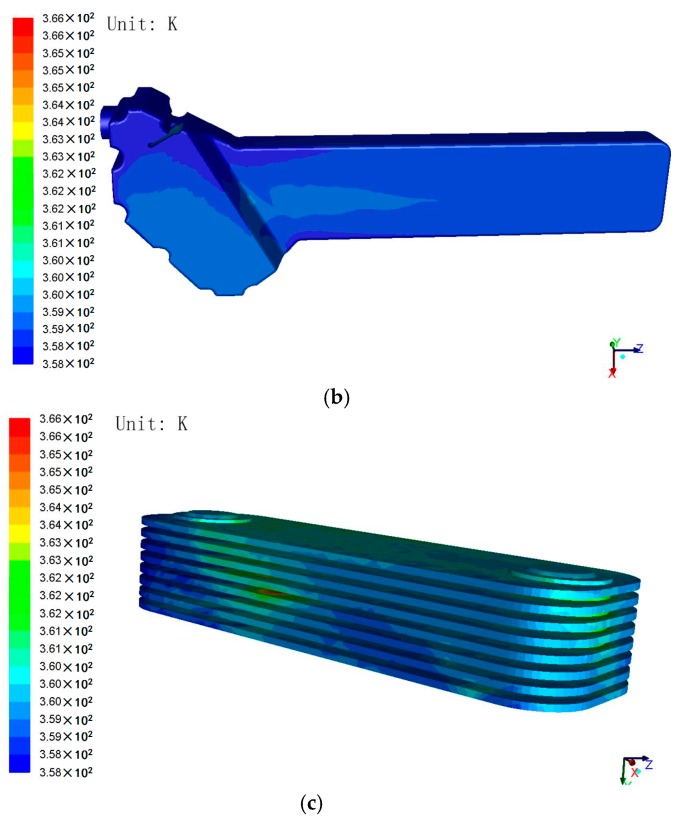
The CFD results of the oil cooling system, (**a**) the pressure distribution and (**b**) the temperature distribution of the water cavity, and (**c**) the temperature distribution of the oil cooler.

The CFD results of the oil cooling system are shown in Figure 11. The pressure and temperature distributions are used as the loading condition of the oil cooler cover in the following structural analysis.

### 4.2. Static Analysis and Results

**Figure 12:**
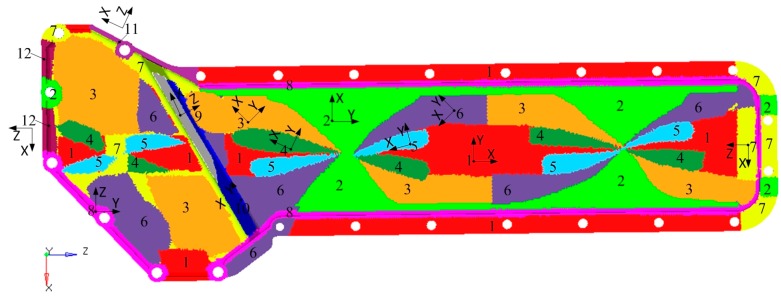
FE models with different material properties. The different numbers indicate the different groups and the different groups have the corresponding colors. The blocks with the same number and the same color are in the same group.

**Figure 13:**
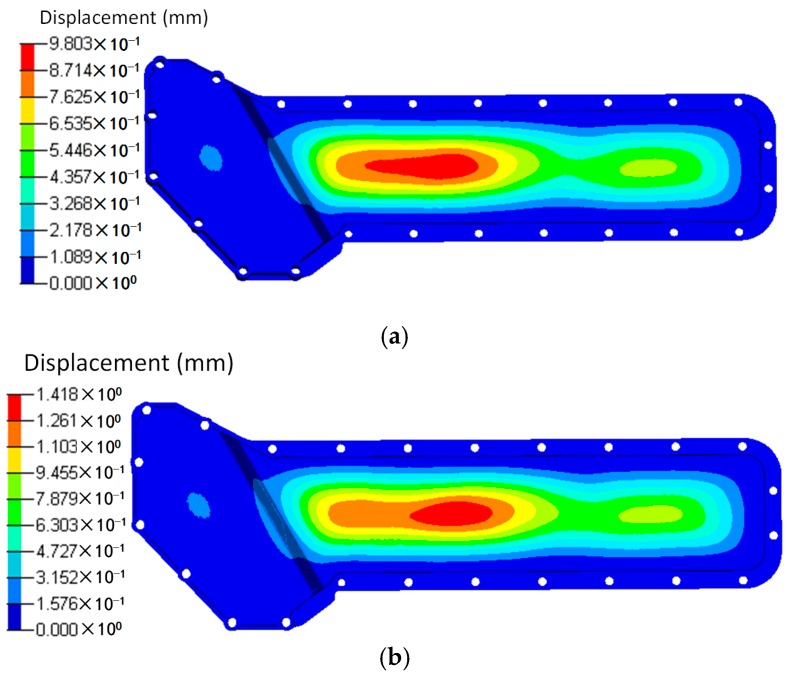
Displacements of (**a**) the isotropic and (**b**) the orthotropic oil cooler covers under distributed pressure.

**Figure 14:**
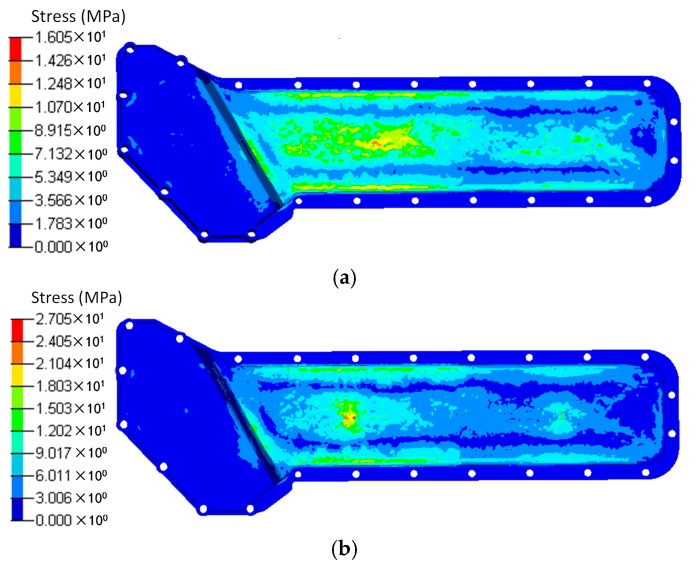
Stress distributions of (**a**) the isotropic and (**b**) the orthotropic oil cooler covers under distributed pressure.

Based on the injection modelling results, the FE model of the oil cooler cover was divided into 12 groups consisted of 52 blocks according to the fiber orientation distribution, as shown in Figure 12. The fiber orientation varies with different groups. Different blocks in the same group have the same fiber orientation. Using local coordinates, the fiber orientation in each group was defined, and the material properties were given according to the stiffness matrix (Equation (3)). The total fiber volume fractions were the same in both cases. In the simulation, the screw holes of the oil cooler cover were constrained in all the six DOFs. The pressure of the water cavity was mapped onto the inner face of the oil cooler cover. The calculation results under pressure are shown in Figure 13 and Figure 14 respectively.

**Figure 15:**
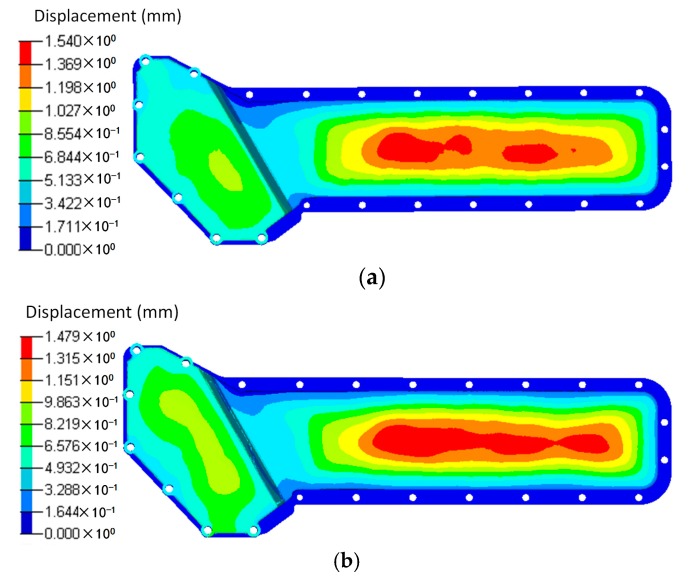
Displacements of (**a**) the isotropic and (**b**) the orthotropic oil cooler covers under distributed temperature difference.

Seen from Figure 13, the deformation distributions of the two models with different materials are similar. The maximum deformation appears at the center of the lower part of the cover. However, the maximum deformation of the orthogonal anisotropic material is about 1.5 times of that of the isotropic material, and the maximum deformation zone of the former is smaller than that of the latter. As shown in Figure 9, the fiber orientation of the maximum deformation area is along the horizontal direction which means the normal modulus is smaller, so that the peak deformation of the orthogonal anisotropic material is larger than that of the isotropic one. As shown in Figure 15, the stress distributions of the two models are very similar, and the stress concentrates at the left center part of the lower cover. However, due to the elastic modulus of the orthogonal anisotropic model, along the horizontal direction is larger than that of the isotropic model, the corresponding maximum stress of the former (27.05 MPa) is about 40% larger than that of the latter (16.05 MPa).

**Figure 16:**
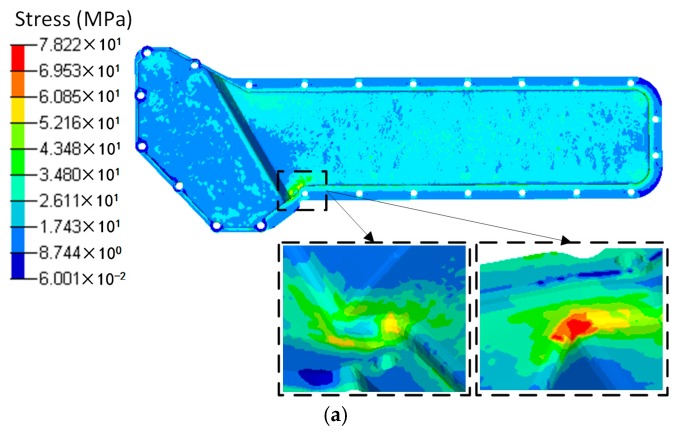

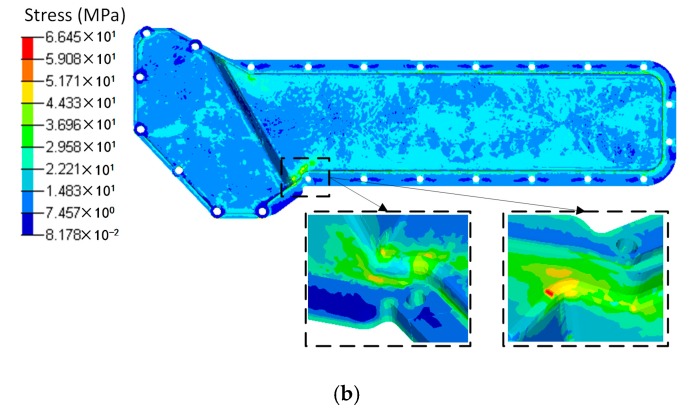
Stress distributions of (**a**) the isotropic and (**b**) the orthotropic oil cooler covers under distributed temperature difference.

Based on the temperature distribution obtained from the CFD analysis, the thermal deformation and thermal stress of the oil cooler covers were analyzed and are shown in Figure 15 and Figure 16 respectively. It can be seen that both the distribution and the peak value of the deformation under the same temperature difference are close between the orthogonal anisotropic and the isotropic models. The maximum deformation zone locates at the center of the lower part of the cover, and the maximum values are 1.54 mm for the isotropic model and 1.48 mm for the orthogonal anisotropic model. For the case of thermal stress, the peak values are 78.22 MPa for the isotropic model and 66.45 MPa for the orthogonal anisotropic model and the maximum stress locates at the lower corner between the higher and the lower parts of the cover surface. Although the differences between the two models are smaller than that of the pressure loaded case, the model with orthogonal anisotropic material shows a better thermal performance than that of the isotropic material in terms of lower peak thermal stress and smaller stress concentration area.

**Figure 17:**
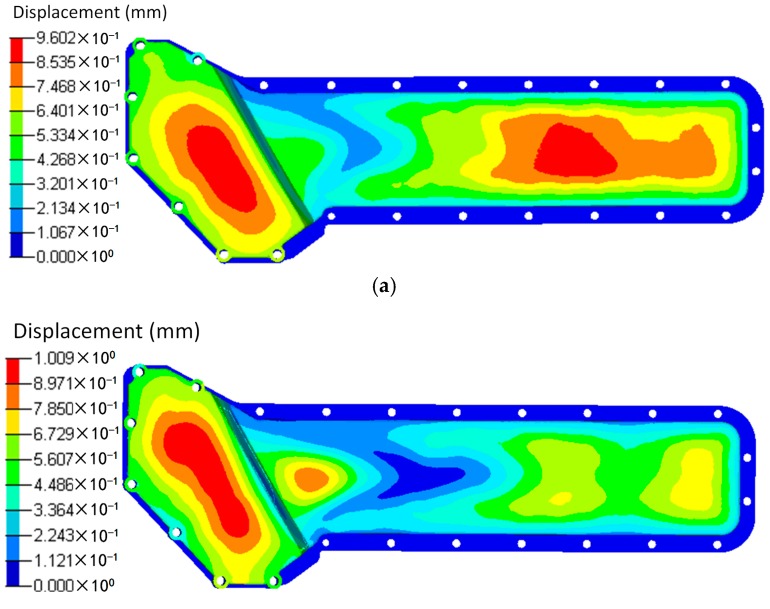
Displacements of (**a**) the isotropic and (**b**) the orthotropic oil cooler covers under distributed pressure and temperature difference.

**Figure 18:**
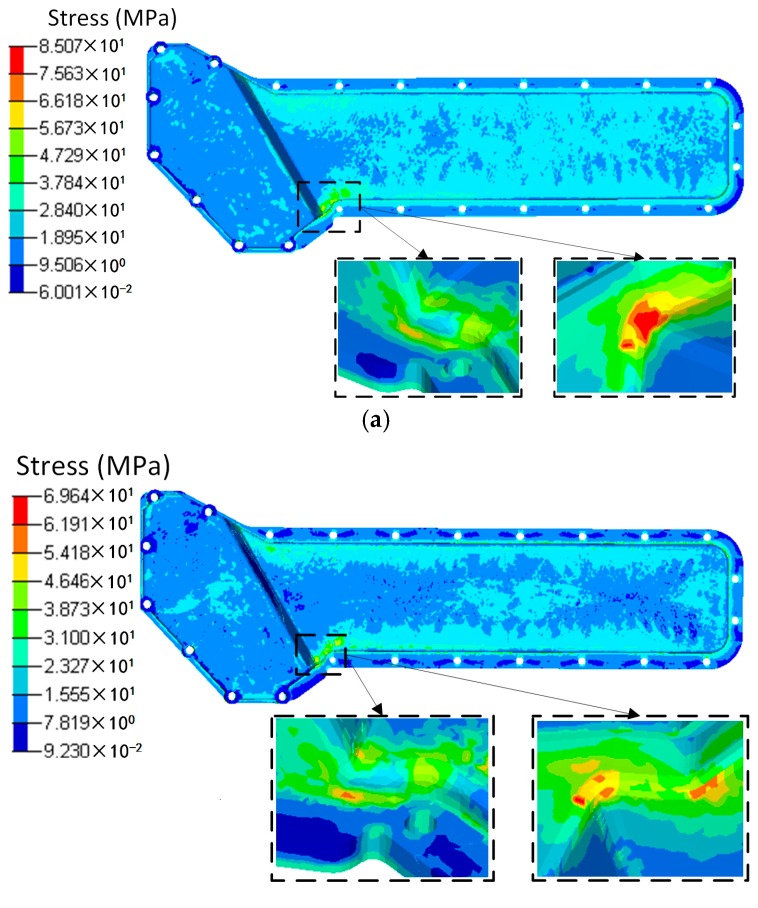
Stress distributions of (**a**) the isotropic and (**b**) the orthotropic oil cooler covers under distributed pressure and temperature difference.

To comprehensively analyze the static response of the plastic cover under both pressure and temperature loads, a coupled analysis was carried out and the deformation and stress results are shown in Figure 17 and Figure 18. As discussed above, the maximum deformation under individual load locates at the center of the lower part of the oil cover no matter whether the load is pressure or temperature difference. However, under the load combining pressure and temperature, the deformation is totally different. In the isotropic material case, two parts of the cover, the centers of both the higher and lower surface, have maximum deformation. In addition, the maximum deformation (0.96 mm) is lower than that of the single loaded cases (0.98 mm under pressure loading and 1.54 mm under temperature loading). In case of the orthogonal anisotropic model, the center of the lower part of the cover is no longer the area of maximum deformation. Instead, the center of the higher part of the cover becomes the maximum deformation zone. Apart from that, the area on the lower side close to the boundary between the higher and lower parts has a relatively high deformation. In terms of the maximum deformation value, the case under coupled loads (1 mm) is the lowest compared to those only under the pressure (1.41 mm) and the temperature (1.47 mm) loadings. Unlike the deformation, the stress distribution under the coupled pressure and temperature is similar to the single loaded cases in which the lower corner between the higher and lower parts of the cover has the peak stress. Under coupled loads, the maximum stress of the orthogonal anisotropic model (69.6 MPa) is smaller than that of the isotropic model (85.1 MPa), which is similar to the case under thermal load. In addition, the peak stress in the coupled loading case is the largest among the three loading conditions. It can be found that it is better to analyze the static response under a coupled loading condition when the component is supposed to serve under more than one kind of load. Moreover, fiber orientation plays an important part in the static performance of a plastic component. In other words, the model with an orthogonal anisotropic material shows different responses of stress and deformation compared to the model with an isotropic material which may strongly affect the product design and test.

**Figure 19:**
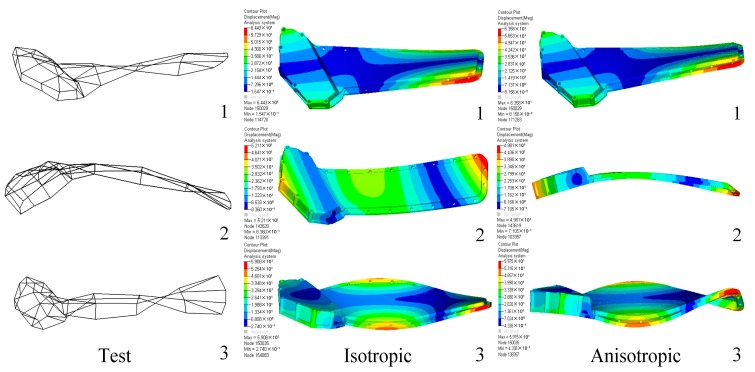
Comparisons of mode shapes of the oil cooler cover between tested and simulated results.

To validate the proposed simulation method of modeling a plastic component with an orthogonal anisotropic material and also to compare with the isotropic material model, a modal test of the plastic oil cooler cover was carried out and the modal analysis was also carried out using the two FE models. The first five order modal frequencies are listed in Table 3, and the first three order modes are shown in Figure 19. It can be seen from the comparison in Table 3 that the modal frequencies predicted by the orthogonal anisotropic model (maximum deviation of 6.95%) are much closer to the tested data than that given by the isotropic model (minimum deviation of 8.64%) in all corresponding orders. In other words, to use the proposed orthogonal anisotropic model can give much more realistic results compared to the general isotropic model.

### 4.3. Dynamic Analysis and Results

**Figure 20:**
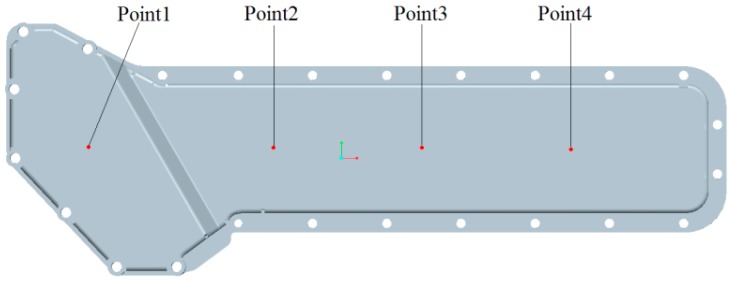
Sketch of the response points on the oil cooler cover.

**Figure 21:**
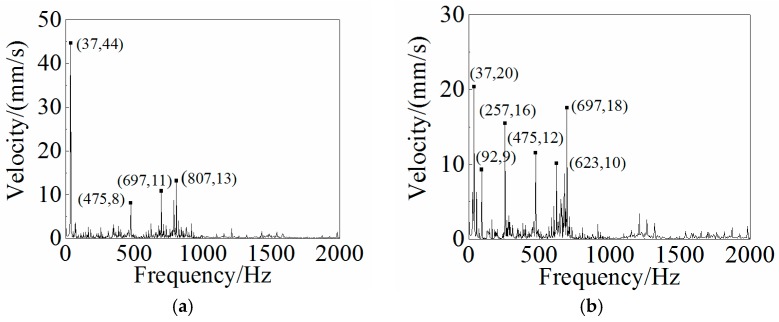
Frequency responses of (**a**) the isotropic and (**b**) the orthotropic oil cooler covers at Point 1 of Figure 20.

**Figure 22:**
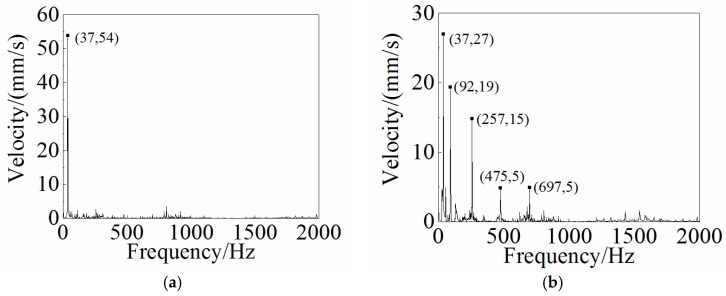
Frequency responses of (**a**) the isotropic and (**b**) the orthotropic oil cooler covers at Point 2 of Figure 20.

**Figure 23:**
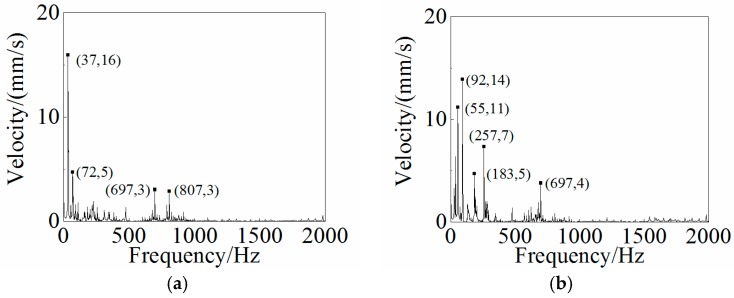
Frequency responses of (**a**) the isotropic and (**b**) the orthotropic oil cooler covers at Point 3 of Figure 20.

**Figure 24:**
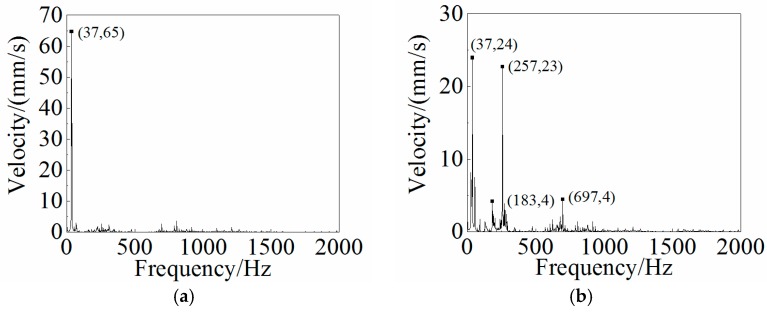
Frequency responses of (**a**) the isotropic and (**b**) the orthotropic oil cooler covers at Point 4 of Figure 20.

Other than the static response to the excitation, the dynamic response analysis of the plastic oil cooler cover using isotropic and orthogonal anisotropic modes was conducted. Using both experimental and simulation methods, the dynamic responses (vibration and acoustic) were obtained under real operating conditions of the oil cooler cover. To prepare the excitation of the dynamic analysis, an inline six-cylinder diesel engine was tested at 2,200 rpm speed. The acceleration at the bolts of the oil cooler cover was recorded to be the input in the following simulation. In the vibration calculation, four response positions (shown in Figure 20) were chosen for the comparison of the dynamic performance between the isotropic and the orthogonal anisotropic models and the results are given in Figure 21, Figure 22, Figure 23 and Figure 24. It can be seen that the vibration responses of the two models are distinctly different in both peak velocity and frequency content. In all the response positions, the peak vibration amplitude frequencies are the same in the two models which is 37 Hz, but the velocity amplitude of the orthogonal anisotropic model (20 mm/s) is far smaller than that of the isotropic model (44 mm/s) except for the point 3. Even for the point 3, although the peak amplitudes of the two models are similar to each other, the orthogonal anisotropic model still gives a smaller result. For other frequencies, the orthogonal anisotropic model show much richer content than the isotropic model, such as the peaks at the 1.5-octave (55 Hz), the 2.5-octave (92 Hz), and the 7-octave (257 Hz) are more apparent than the results obtained by the isotropic model. The differences mean that the vibration transfer functions and the overall stiffness are both changed by changing the material properties.

**Figure 25:**
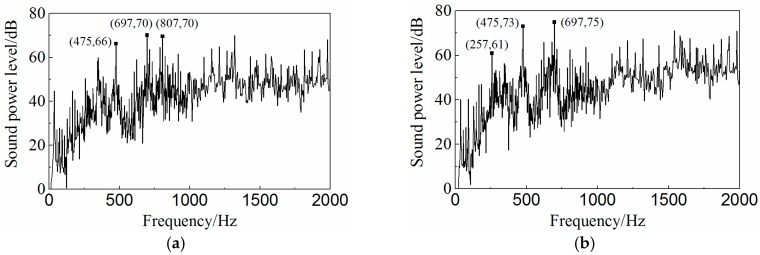
Predictions of noise of (**a**) the isotropic and (**b**) the orthotropic oil cooler covers.

Based on the vibration results, the radiated noise of the oil cooler cover with different fiber orientations was predicted under the same operating condition as the vibration analysis. The acoustic model (195,804 surface elements) was constructed using LMS Virtual.Lab 13.0 (Siemens PLM Software, Plano, TX, USA) consisting of the acoustics mesh, the symmetry plane, and the acoustic field. The symmetry plane and the acoustic field were constructed automatically in the acoustic software, which were then used to simulate the ground reflection of the noise and the atmospheric sound propagation. The radiated noise was predicted using the direct boundary element method, and the sound power spectra are shown in Figure 25. The overall sound power level of the isotropic cover is 80.33 dB and that of the orthogonal anisotropic one is 83.64 dB which is about 3 dB higher than the former. The difference in the frequency distribution of the sound power between the two models is smaller than that in the vibration velocity case. But at high frequency, the noise of the orthogonal anisotropic model is still higher than that obtained from the isotropic model.

## 5. Conclusions

In this study, a simulation method for modeling the orthogonal anisotropic plastic component was proposed and compared with the general isotropic model. A simple numerical example of a square plate was studied using isotropic and orthogonal anisotropic materials in order to show the difference between the two materials in terms of static performances. After that, a comprehensive performance comparison between the two materials was conducted based on a plastic diesel engine oil cooler cover made by glass fiber reinforced PA66. The proposed method was validated by a modal test, and a series of static and dynamic responses were calculated and compared between the proposed orthogonal anisotropic model and the general isotropic model. Main conclusions can be drawn as follows:

Due to the material properties varying from the horizontal direction to the normal direction, the stress and deformation distributions of the orthogonal anisotropic plate are axisymmetric while those of the isotropic plate are centrally symmetrical. At the same time the peak stress and the deformation are different between the two models.

Based on the modal test of the plastic oil cooler cover, the proposed model with orthogonal anisotropic material was validated to provide more accurate predictions compared to the general isotropic modeling.

The static simulations of the oil cooler cover under a steady pressure-temperature coupled load show big differences from those under single pressure or temperature load. Under the coupled loading condition, although the maximum deformation between the two models is similar, the maximum deformation location of the orthogonal anisotropic model is at the center of the higher part of the cover, while that of the isotropic model also appears at the center of the lower part. On the contrary, the stress distributions between the two models are almost the same, but the peak stress value of the orthogonal anisotropic model is far lower than that of the isotropic one.

Under real operating condition, the vibration responses of the orthogonal anisotropic oil cooler cover are different to that of the isotropic one. In detail, not only the peak vibration velocity of the former is much lower than that of the latter, but also the former model can provide more information in frequency content compared to the latter model. The acoustic response in terms of overall sound power level of the orthogonal anisotropic model is 3 dB smaller than that of the isotropic.

The proposed modeling method using orthogonal anisotropic material could give closer results to reality than the general isotropic model no matter whether for static or dynamic performances. In other words, the proposed modeling method might be more appropriate in plastic product design and test procedures.

## Figures and Tables

**Table 1:** Properties of PA66 (glass fiber reinforced polyamide 66) (GF 30%).

Properties	Values
Material structure	Crystalline
Glass fiber	30%
Solid density (g/cm^3^)	1.36
Melt density (g/cm^3^)	1.16
*E*_1_ (MPa)	10,300
*E*_2_ *E*_3_ (MPa)	6,800
μ_12_,μ_13_	0.4
*G*_12_ (MPa)	1,910
*α*_1_ (1/K)	3 × 10^−5^
*α*_2_ *α*_3_ (1/K)	9 × 10^−5^
Ejection temperature (°C)	185
Recommended mold temperature (°C)	90
Recommended melt temperature (°C)	300

Note: subscript 1 indicates the direction along the fiber orientation, and subscripts 2 and 3 indicate the directions normal to the fiber orientation.

**Table 2:** Comparisons of free modal frequencies between the isotropic and the anisotropic plates.

Order	Isotropic (Hz)	Anisotropic (Hz)
1	31.29	24.90
2	45.92	44.26
3	60.59	60.51
4	81.93	68.53
5	81.93	75.79

**Table 3:** Comparisons of modal frequencies of the oil cooler cover between tested and simulated results.

Order	Test	Isotropic	Deviation (%)	Anisotropic	Deviation (%)
1	32.78	40.14	22.45	34.15	4.18
2	47.72	55.05	15.36	51.04	6.95
3	98.95	109.90	11.07	96.25	2.73
4	122.02	137.10	12.36	126.25	3.46
5	169.37	184.00	8.64	165.50	2.28

## Appendix

The uncoupled stress–strain relationship can be defined as:

(A1) $${\left\{\varepsilon \right\}}_{m}={\left[C\right]}_{m}{\left\{\sigma \right\}}_{m}$$

(A2) $${\left\{\sigma \right\}}_{m}={\left[C\right]}_{m}^{-1}{\left\{\varepsilon \right\}}_{m}={\left[D\right]}_{m}{\left\{\varepsilon \right\}}_{m}$$

(A3) $${\left\{\varepsilon \right\}}_{m}={\left[\begin{array}{cccccc} \varepsilon_{11} & \varepsilon_{22} & \varepsilon_{33} & \gamma_{12} & \gamma_{23} & \gamma_{31} \end{array}\right]}^{T}$$

(A4) $${\left\{\sigma \right\}}_{m}={\left[\begin{array}{cccccc} \sigma_{11} & \sigma_{22} & \sigma_{33} & \tau_{12} & \tau_{23} & \tau_{31} \end{array}\right]}^{T}$$

where *ε* is the strain, *σ* is the stress; *ε*_ii_ is the tensile strain, *γ*_ij_ is the shear strain, *σ*_ii_ is the tensile stress, and *τ*_ij_ is the shear stress. The flexibility matrix [*C*]_m_ can be given as:

(A5) $${\left[C\right]}_{m}=\left[\begin{array}{cccccc} 1/E_{1} & -\mu_{21}/E_{1} & -\mu_{31}/E_{1} & 0 & 0 & 0\\ -\mu_{12}/E_{1} & 1/E_{2} & -\mu_{32}/E_{2} & 0 & 0 & 0\\ -\mu_{13}/E_{1} & -\mu_{23}/E_{2} & 1/E_{3} & 0 & 0 & 0\\ 0 & 0 & 0 & 1/G_{23} & 0 & 0\\ 0 & 0 & 0 & 0 & 1/G_{13} & 0\\ 0 & 0 & 0 & 0 & 0 & 1/G_{12} \end{array}\right]$$

where *E*_i_ is the tensile elastic modulus, *G*_ij_ is the shear elastic modulus, *μ*_ij_ is Poisson’s ratio. As the elastic matrix of the orthotropic material is symmetrical, *E*_1_*μ*_21_ = *E*_2_*μ*_12_, *E*_2_*μ*_23_ = *E*_3_*μ*_23_ and *E*_3_*μ*_13_ = *E*_1_*μ*_31_, only nine constants in Equation (A5) are independent.

By reversing the flexibility matrix, the stiffness matrix can be obtained in which the constants are given as:

(A6) $$\{\begin{array}{c} d_{11}=\frac{1-\mu_{23}\mu_{32}}{E_{2}E_{3}\Delta }\\ d_{22}=\frac{1-\mu_{13}\mu_{31}}{E_{1}E_{3}\Delta }\\ d_{33}=\frac{1-\mu_{12}\mu_{21}}{E_{1}E_{2}\Delta }\\ d_{12}=d_{21}=\frac{\mu_{21}+\mu_{31}\mu_{23}}{E_{2}E_{3}\Delta }=\frac{\mu_{12}+\mu_{13}\mu_{32}}{E_{1}E_{3}\Delta }\\ d_{23}=d_{32}=\frac{\mu_{32}+\mu_{12}\mu_{31}}{E_{1}E_{3}\Delta }=\frac{\mu_{23}+\mu_{21}\mu_{13}}{E_{2}E_{3}\Delta }\\ d_{31}=d_{13}=\frac{\mu_{13}+\mu_{12}\mu_{23}}{E_{1}E_{2}\Delta }=\frac{\mu_{31}+\mu_{21}\mu_{32}}{E_{2}E_{3}\Delta }\\ d_{44}=G_{12}\\ d_{55}=G_{23}\\ d_{66}=G_{31} \end{array}$$

where Δ = (1 − *μ*_12_*μ*_21_ − *μ*_23_*μ*_32_ − *μ*_3_*μ*_13_-2*μ*_12_*μ*_23_*μ*_31_)/*E*_1_*E*_2_*E*_3_.

A transversely isotropic material has symmetric properties about the axis normal to a plane of isotropy [40]. The material properties are the same in all directions within the transverse plane. The unidirectional composite material is transversely isotropic and the plane normal to the fiber orientation can be considered as the transverse plane. Let the axis along the fiber orientation be the *x*_1_ direction, and the other two axes on the transvers plane be the *x*_2_ and *x*_3_ directions. The relationships of the elastic modular, the Poisson’s ratio, and the shear modular between different directions can be obtained as:

(A7) $$\{\begin{array}{c} E_{2}=E_{3}\\ \mu_{12}=\mu_{13},\mu_{21}=\mu_{31},\mu_{32}=\mu_{23}\\ G_{13}=G_{12} \end{array}$$

Accordingly the number of independent constants in the flexibility matrix can be reduced to five and given as:

(A8) $${\left[C\right]}_{m}=\left[\begin{array}{cccccc} 1/E_{1} & -\mu_{12}/E_{1} & -\mu_{12}/E_{1} & 0 & 0 & 0\\ -\mu_{12}/E_{1} & 1/E_{2} & -\mu_{23}/E_{2} & 0 & 0 & 0\\ -\mu_{12}/E_{1} & -\mu_{23}/E_{2} & 1/E_{2} & 0 & 0 & 0\\ 0 & 0 & 0 & 1/G_{23} & 0 & 0\\ 0 & 0 & 0 & 0 & 1/G_{12} & 0\\ 0 & 0 & 0 & 0 & 0 & 1/G_{12} \end{array}\right]$$

where *G*_23_ = *E*_22_/[2(1 + *μ*_23_)].
